# The Development of the Umbilical Vein and Its Anatomical and Clinical Significance

**DOI:** 10.7759/cureus.79712

**Published:** 2025-02-26

**Authors:** Alexandros Kozadinos, Adam Mylonakis, Filippos Bekos, Nikolaos Kydonakis, Georgios Korovesis, Pagona Kastanaki, Markos Despotidis, Dimosthenis Chrysikos, Theodore Troupis

**Affiliations:** 1 First Department of Surgery, Laiko General Hospital, National and Kapodistrian University of Athens, Athens, GRC; 2 Department of Anatomy, Medical School, National and Kapodistrian University of Athens, Athens, GRC

**Keywords:** anatomical variations of liver vasculature, development of umbilical vein, liver surgery, umbilical vein and surgery, umbilical vein variations

## Abstract

The umbilical vein is one of the most essential vessels in the human embryo. Anatomical structures though may vary in several cases. During the fourth and eighth weeks of gestation, the umbilical cord is formed. Initially, two umbilical arteries and veins exist. During development, the obliteration of the right umbilical vein occurs. The fetus and its liver receive macronutrients and oxygen from the placenta via the umbilical vein, which primarily supplies the left lobe of the liver before branching into the left portal vein and the ductus venosus. The ductus venosus directs blood from the umbilical vein directly into the systemic circulation through the inferior vena cava and right atrium, bypassing the fetal liver. In some cases, variations are observed. Disorders of the umbilical veins may involve the persistence of embryological structures, abnormal insertion or course, and the presence of supernumerary vessels. For example, the persistence of the right umbilical vein, duplication of the umbilical vein, and umbilical vein varix are some important variations to acknowledge in order to be able to understand the potential outcomes of the newborn. The majority of venous system anomalies are rare, and some may remain completely asymptomatic. Different forms of umbilical cord abnormalities, however, may be potentially fatal or pose a serious threat to fetal health. Therefore, clinically, early detection of these malformations is highly important in order to make a proper diagnosis and management of care. The aim of this study is to acknowledge the different types of umbilical vein variations through its development and its relation with liver parenchyma in order to achieve a better understanding and planning in surgical interventions. An advanced review search of the literature was undertaken. The literature review was conducted using the search engine of the PubMed database and Google Scholar. The years included in data collection were 1960-2022. All articles that met the inclusion criteria were taken under consideration.

## Introduction and background

Introduction

The study of the human body and human physiology, even before birth, unlocks a new whole horizon of an in-depth understanding of the human body's function. The location of each anatomic element serves a purpose and can be explained. After understanding the purpose and derivation of anatomical structures, we can then elaborate on several relationships within the body and plan further interventions in order to achieve benefits for human health. As in all things, structural variations and anomalies sometimes arise that must be studied and understood in order to be able to handle them accordingly. Not observing such variations can prove catastrophic for the human body while attempting even minor interventions. Embryology, anatomy, and surgical anatomy work hand in hand in such cases in order to provide state-of-the-art surgical treatments for patients who might suffer from a variety of pathologies. More specifically, the parenchyma of the liver, an organ very abundant in blood supply, and for many considered to involve the highest degree of difficulty in terms of surgical intervention, must be studied thoroughly. One must achieve a high grade of knowledge of topography, as well as the development of this organ, in order to plan surgical interventions confidently and in good time. The main focus of this study is to establish our knowledge on the variations of the umbilical vein as well as their outcomes, in order to apply our surgical capabilities in such patients. The development and the variations of the umbilical vein, the main vessel of maternal blood inflow during the development of the fetus, will be mentioned in order to have a great understanding of the vessels' anatomy. Furthermore, a drastic change in the functional vessel into one of the main suspensory ligaments of the liver parenchyma will be studied, and its relationship to modern surgery will be discussed.

Methods

A comprehensive literature search was conducted using the PubMed search engine and Google Scholar database to identify relevant studies on the anatomical variations and physiological characteristics of the umbilical vein, as well as its implications in hepatic surgery. The search strategy included the following key terms: "Umbilical vein AND anatomical variations" (31 results), "Physiology of Umbilical vein AND variations" (43 results), and "Umbilical vein AND hepatic surgery" (47 results).

Inclusion Criteria

The review included observational studies, systematic reviews, case reports/series, and renowned published medical literature books investigating the development, anatomy, and variations of the umbilical vein. The retrieved articles were systematically reviewed to ensure relevance to the scope of the study. Studies were considered eligible for inclusion if they strictly focused on human subjects and were published between 1960 and 2022. References from the initially selected articles were further examined to incorporate additional relevant literature, particularly those providing fundamental knowledge on liver anatomy, umbilical vein physiology, and their clinical significance in hepatic surgery. This process allowed for a broader synthesis of information, enhancing the depth of our narrative review.

Exclusion Criteria

Studies that focused primarily on animal models, those published before 1960 or after 2022, and cases reporting congenital diseases with concomitant vascular anomalies were excluded. Articles with incomplete or insufficient data on the umbilical vein's variations and its surgical relevance were also excluded to maintain the quality and specificity of the review. Additionally, studies lacking full-text availability as well as non-peer-reviewed sources were not taken under consideration. 

This systematic approach ensured a comprehensive evaluation of the existing literature, providing a well-rounded perspective on the anatomical and physiological aspects of the umbilical vein and its variations.

## Review

Results

Umbilical Cord Embryology and the Development of Umbilical Veins

The primitive umbilical cord arises between the fourth and eighth weeks of gestation (calculated from the first day of the last menstrual period) as the expanding amnion envelops tissue from the body stalk, the umbilical coelom, and the omphalomesenteric duct. By the end of the fifth week of gestation, blood flow within the umbilical cord is established [1]. The body stalk contains two umbilical arteries, two umbilical veins, and the allantois. Initially, the left and right umbilical arteries are caudal extensions of the primitive dorsal aortae. However, after multiple developmental changes, they ultimately originate from the internal iliac arteries [2]. Following birth, the proximal segments of the intra-abdominal umbilical arteries develop into the internal iliac and superior vesical arteries, while the distal segments regress to form the medial umbilical ligaments [3]. The umbilical veins arise from a network of venules that drain the extra-embryonic allantois [2]. The right umbilical vein regresses by the end of the sixth week of gestation [4], leaving only the left umbilical vein to persist. The ligamentum teres is a hepatic remnant formed when the intra-abdominal umbilical vein is obliterated at birth [3]. Variations in the ligamentum teres anatomy and the quadrate lobe are rare anomalies of the developing liver, usually noted during autopsies, surgeries, and cadaveric dissections for routine medical training [5]. The fissure of the ligamentum teres may have variable depth and ascend backward from its notch on the inferior border to the left end of the fissure for the ligamentum venosum. The allantois originates as a diverticulum of the yolk sac, extending from the early fetal bladder into the body stalk, and plays a role in the development of the umbilical vessels. Its regression occurs between the sixth and eighth weeks of gestation, with the remnant positioned within the cord and between the two umbilical arteries. The intra-abdominal remnant of the allantois transforms into a thick tubular structure known as the urachus or median umbilical ligament [6]. Two umbilical arteries, one umbilical vein, and the remnant of the allantois are all embedded in Wharton's jelly and enclosed by a single layer of amnion in a fully formed umbilical cord. 

**Figure 1:**
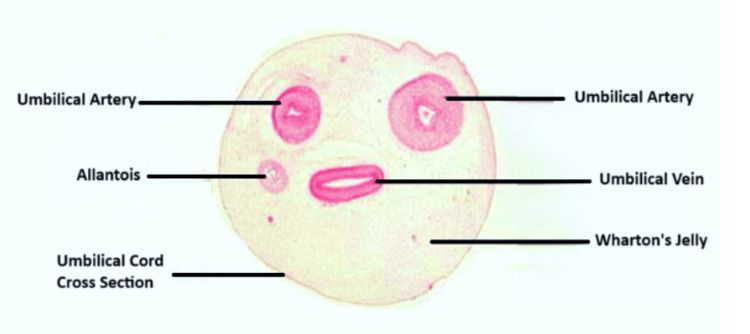
Cross section of the umbilical cord under the microscope Recreated based on image from the original source: https://en.wikipedia.org/wiki/Cord_lining#/media/File:Cross_section_of_the_umbilical_cord.jpg

Figure 1 depicts the cross section of the umbilical cord under the microscope.

The final length of the umbilical cord ranges from 50 cm to 60 cm [7], while the physiological cord length can range from 30 cm to 100 cm (less than 30 cm is considered short) [8]. Prolapse, looping of the cord around the fetal neck, entanglement, distress, and fetal demise [8] have been associated with extremely long cords. In contrast, very short umbilical cords may be linked to delayed fetal descent, premature placental separation [9], fetal growth restriction, congenital abnormalities, fetal distress, and even fetal demise [8]. A study with 368 healthy pregnant women showed that the average umbilical cord diameter ranged from 3.19±0.40 mm at the 10th week of gestation to 16.72±2.57 mm at the 33rd to 35th weeks, and it then decreased to 14.42±1.50 mm at the 42nd week of gestation [7]. The decrease in cord diameter as the pregnancy progresses is due to the decline in the Wharton's jelly water content [10], a connective tissue rich in mucous components that surrounds the umbilical arteries. The umbilical vein's diameter within the umbilical cord increases from 4.1 mm at the 20th week to 8.3 mm at the 38th week of gestation [11]. Another study showed an increase in the cross-sectional area of the umbilical cord vein from 28 mm^2^ at the 24th week of gestation to approximately 58 mm^2^ between 34 and 38 weeks, followed by a slight reduction from the 39th week [12]. The reduction of the diameter of the vessel by approximately 1 mm between the placental and fetal ends is another variation in the umbilical cord vein [13]. The umbilical vein in the umbilical cord is approximately 30% larger in cross-sectional area than the combined areas of the arteries, resulting in a velocity that is about half that of each artery [14]. The velocity of blood flow in the umbilical vein ranges from 10 to 22 cm/s [15]. The umbilical vein carries oxygenated blood to the fetal heart, while the umbilical arteries return deoxygenated blood to the placenta, a pattern that contrasts with the typical functions of veins and arteries in the rest of the fetal circulatory system. Unlike other arteries, the umbilical arteries lack an internal and external elastic lamina, with their adventitia replaced by mucous connective tissue. The umbilical vein, however, features a thickened muscularis layer composed of interwoven circular, longitudinal, and oblique smooth muscle fibers, as well as an internal elastic lamina [16].

The placenta maintains a huge amount of blood, which is then transported to the fetus. "The volume of blood going from the fetus to the placenta approximately equals the amount flowing from the placenta to the fetus" [16], rendering the fetus a closed system. The following methods describe the movement of oxygenated blood from the placenta to the fetus: First, the pressure in the umbilical vein increases from 4.5 mmHg at the 18th week of gestation to 6 mmHg by the end of pregnancy [15]. Additionally, the blood pressure within the umbilical vein is higher than that in the fetal inferior vena cava [15]. This pressure gradient is influenced by at least two mechanisms: physiological fetal heart contractions generate a pressure gradient between the atria and ventricles, decreasing venous preload and facilitating the flow of blood in the umbilical vein toward the heart [17], and variations in abdominal and thoracic cavity pressures induced by fetal breathing movements create a pressure gradient between the umbilical vein and the ductus venosus, increasing blood velocity in the umbilical vein during inspiration [17]. Second, changes in the passive pressure within the umbilical vein occur due to the longitudinal distortion of the arteries with each fetal heartbeat. The pressure peaks in the umbilical artery and vein are 180° out of phase, resulting in the cumulative effect of multiple minor pressure variations along the length of the cord, which facilitates blood movement through the umbilical vein [14].

Anatomy and Function of Umbilical Veins in the Embryo

The fetal liver is pivotal in regulating fetal energy homeostasis and growth by metabolizing energy-providing nutrients. It synthesizes lipids, stores glycogen, and serves as a source of various fetal growth factors, thereby supporting the energy demands and developmental processes of the fetus [18]. The fetus and its liver receive macronutrients and oxygen from the placenta via the umbilical vein, which primarily supplies the left lobe of the liver before branching into the left portal vein and the ductus venosus. The ductus venosus directs blood from the umbilical vein directly into the systemic circulation through the inferior vena cava and right atrium, bypassing the fetal liver. Most of the blood from the ductus venosus flows to the left side of the heart through the foramen ovale after reaching the right atrium, to supply essential fetal organs such as the heart and brain [15]. The venous intersection, where blood from the umbilical vein either is distributed to the right liver lobe or bypasses the liver via the ductus venosus, plays a crucial role in understanding the fetal distribution of energy-providing nutrients. This process is essential for investigating how incoming nutrients are allocated to support fetal development and growth. 

**Figure 2:**
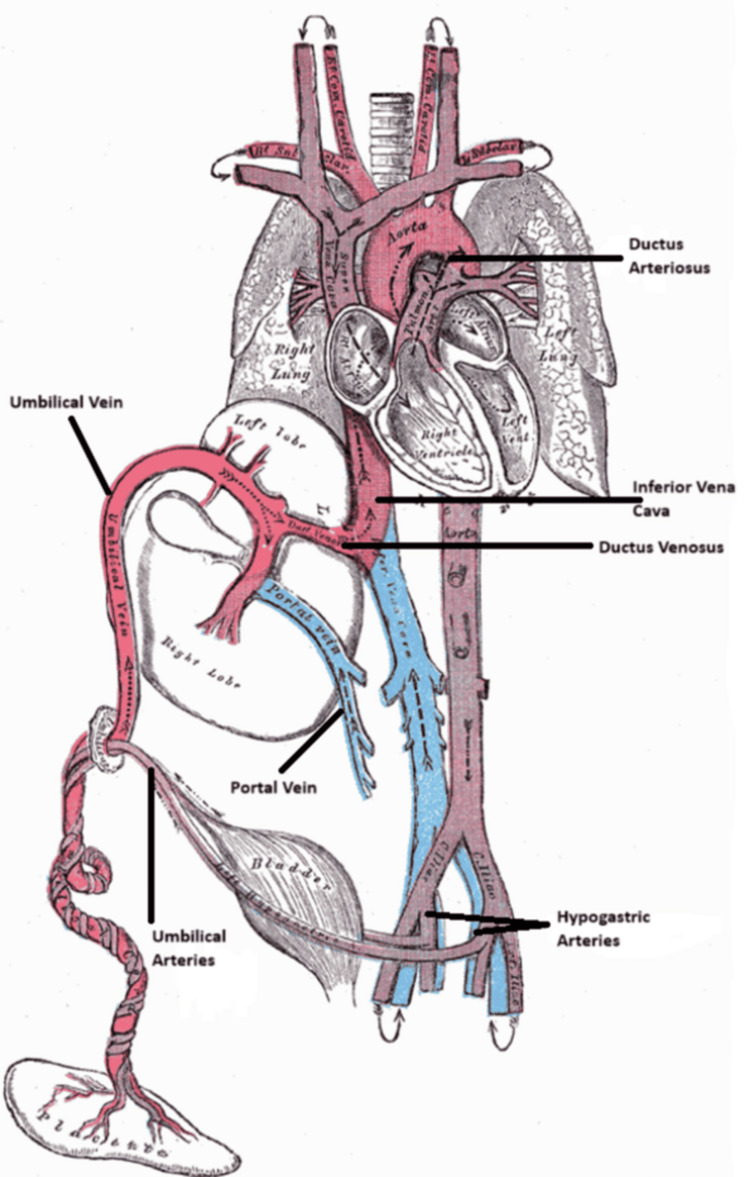
Schematic illustration of fetal circulation Recreated based on image from the original source: https://en.wikipedia.org/wiki/Fetal_circulation#/media/File:Fetal_circulation.png

Figure 2 depicts the schematic illustration of fetal circulation.

There is evidence to suggest that the distribution of umbilical blood through the liver is a key mechanism regulating fetal energy metabolism and, consequently, fetal development. This hypothesis is supported by positive correlations observed between liver blood flow, growth, and fat accretion during the last trimester, as demonstrated in studies based on human ultrasound examinations [19].

Common and Uncommon Anatomic Variations of Umbilical Veins

The umbilical vein has right and left branches that originate from the placenta and emerge from the chorion, passing via the umbilical cord into the embryo's body and entering the sinus venosus through the primordial septum transversum during early embryonic development. Under normal circumstances, the right umbilical vein undergoes atresia starting in the fourth embryonic week and disappears by the seventh week. Similarly, the portion of the left umbilical vein proximal to the heart, located between the liver and the sinus venosus, degenerates. However, the segment of the left umbilical vein extending from the umbilicus to the liver persists and connects with the umbilical vein in the umbilical cord. This remaining segment facilitates the return of oxygenated blood from the placenta to the inferior vena cava via the ductus venosus, which forms within the liver [20].

Variations of the umbilical veins can include the abnormal insertion or course, the persistence of embryological structures, and the presence of supernumerary vessels. Most anomalies of the venous system are rare, and some may remain completely asymptomatic. During early development, both umbilical veins are initially connected to the sinus venosus. As mentioned before, the obliteration of the right umbilical vein begins before the fourth week of gestation, and by the seventh week, it typically disappears [21]. The left umbilical vein, which becomes connected to the left portal vein in the fetal liver, subsequently transports all oxygenated blood from the placenta. If the right umbilical vein remains patent, it continues to carry oxygenated blood to the heart. As an intrahepatic supernumerary structure, it may coexist alongside the left umbilical vein [22].

Regarding the abnormal course (i) of the umbilical vein, a few important studies have demonstrated different variations. Usually, in this group of malformations, the patients appear to have symptoms of mechanical intestinal obstruction. Svendsen et al. and Prust and Eskandari [23,24] both demonstrated studies where patients presented with signs and symptoms of ileus, which was found during surgery to be at least partially caused by structures identified as aberrant umbilical veins. These veins traversed the peritoneal cavity without any attachment to or ensheathment by the falciform ligament. In two cases, the aberrant band extended anterior to the transverse colon and small intestine, compressing them against the posterior abdominal wall. This abnormality likely did not originate primarily from the umbilical vein itself but rather from a deficiency in the falciform ligament, which normally provides structural support to the vein. The falciform ligament is a remnant of the ventral mesentery of the foregut, alongside other derivatives such as the lesser omentum and the visceral peritoneum of the liver. These findings suggest that, in the described cases, a portion of the ventral mesentery forming the falciform ligament had regressed, leaving the umbilical vein free and unanchored. Consequently, the relatively mobile intestine could rotate around this sagittally oriented band or become trapped between it and other structures. Abnormal cords or bands in atypical locations are well-documented causes of intestinal obstruction.

Another study by Ricklan et al. [25] demonstrated a rare case involving a 16-year-old patient who exhibited a particularly striking anatomical variation: the umbilical vein running in an abnormally positioned ligamentum teres. This embryological remnant traversed the gallbladder fossa, running to the right of the gallbladder, before terminating in the right branch of the portal vein. Notably, it was not in direct continuity with the ligamentum venosum, which maintained a normal course from the left branch of the portal vein to the left hepatic vein, just proximal to its entry into the inferior vena cava. The fissure for the ligamentum venosum appeared normal, and the ligamentum venosum itself was not patent.

Furthermore, an interesting case of abnormal insertion was reported in a study where a unique case of a stillborn fetus appeared with its umbilical vein bypassing the hepatic parenchyma and coursing itself directly into the right atrium [26]. Similarly, umbilical phlebography in a different case once again revealed the presence of a right umbilical vein draining directly into the inferior vena cava [27]. However, in both of these instances, the patients appeared to have the coexistence of associated abnormalities or life-threatening conditions.

One additional form of altered embryonic development (ii) is the persistence of the right umbilical vein where the left umbilical vein relapses while the right vein remains open. The exact incidence of this lesion has not been determined; however, recent data suggest that a persistent right umbilical vein is more common than previously believed, with an estimated prevalence of one in 250 to one in 1250 pregnancies [28]. The actual incidence of a persistent right umbilical vein is likely higher than reported, as the abnormality can often be overlooked during routine ultrasound examinations, particularly those focused on measuring abdominal circumference. A better diagnosis may be achieved by more effective ultrasound techniques, color Doppler, and three-dimensional ultrasounds [29]. The etiology of a persistent right umbilical vein is not yet known; however, some causes, such as thrombus obstruction, teratogens, or folic acid deficiency, have been reported [28]. Recently, retrospective studies have shown that fetuses with isolated persistent right umbilical vein have a good prognosis [30]. However, persistent right umbilical vein can be associated with the congenital absence of the ductus venosus and other severe fetal malformations. In isolated cases where the ductus venosus is normally connected and the portal system is intact with all its branches, normal hemodynamics can be expected. Nevertheless, close monitoring is essential to detect early signs of hemodynamic decompensation in cases where the ductus venosus is absent [30].

The diagnosis of a persistent right umbilical vein is conducted in a transverse section of the fetal abdomen, and the sonographic criteria include (1) an aberrant course of the portal vein toward the stomach, (2) the fetal gallbladder being medial to the umbilical vein, and (3) the connection of the umbilical vein to the right portal vein instead of the left portal vein.

Another rare abnormality, known as duplication of the umbilical vein (iii), is characterized by an increase in the number of umbilical vessels to four, consisting of two arteries and two veins [22]. Duplication of the umbilical vein occurs when both the left umbilical vein and the right umbilical vein remain open, instead of the right umbilical vein undergoing the typical process of degeneration. In most cases, ultrasonography depicts the right umbilical vein inserting into the liver and linking with the right portal vein, and the left umbilical vein usually combines with the left portal vein and drains into the inferior vena cava through the ductus venosus as a normal course. A better outcome is noticed in patients with isolated duplication of the umbilical vein (intrahepatic persistent right umbilical vein), while after birth, no intervention is needed. Previous studies have indicated that duplication of the umbilical vein may be associated with abnormalities in the cardiovascular, neurological, or facial systems [31]. The prognosis depends on the severity of these associated malformations, and genetic testing is recommended to further evaluate potential underlying anomalies.

Also, one more malformation of the umbilical cord of the fetus which accounts for 4% of abnormalities, with an incidence of 0.4-1.1/1000, is umbilical vein varix (normal direction) [32]. Umbilical vein varix is diagnosed when a segment of the umbilical vein is at least 50% broader than the non-dilated portion, shows a dilatation of ≥9 mm, or has a diameter exceeding 2 standard deviations above the mean value for the corresponding gestational age [33].

Furthermore, during gestational weeks 20-40, the mean inner diameter of the vein in the cord is 3.6-8.2 mm in low-risk fetuses, while the umbilical ring's corresponding diameter is less at 2.8-5.9 mm [34]. A narrower inner diameter of the umbilical vein than the mean diameter for the corresponding gestational weeks and venous blood velocity up to 150-200 cm/s may be diagnostic criteria for umbilical vein constriction. Umbilical vein constriction can lead to pregnancy complications such as intrauterine fetal demise (IUFD), intrauterine growth restriction (IUGR), and oligohydramnios. Umbilical vein constriction is associated with reduced blood flow to the fetus, which, when insufficient to meet the developmental demands, may result in IUGR, fetal hypoxia, acidosis, and eventually IUFD [35]. Early detection of abnormal fetal heart rate patterns through close fetal surveillance plays a crucial role in preventing IUFD [36]. Therefore, careful evaluation of umbilical vein constriction is essential to mitigate the risk of poor perinatal outcomes.

Table 1 depicts the most common anatomical variations of the umbilical vein, summary of diagnosis, and outcomes.

**Table 1:** Most common anatomical variations of the umbilical vein, summary of diagnosis, and outcomes

Anatomical variation	Sonographic findings	Prognosis	Management recommendations
Persistent right umbilical vein [28-30]	Abnormal portal vein course toward the stomach; gallbladder positioned medial to the umbilical vein, umbilical vein connects to the right portal vein instead of the left portal vein	Low risk of complications for isolated cases; prognosis depends on the severity of additional anomalies	Requires detailed fetal anatomical and cardiac evaluation. If findings are normal, no follow-up is necessary. For additional anomalies, consider genetic counseling and invasive testing
Duplication of the umbilical vein [21,31]	Two umbilical veins in the fetal abdomen, draining into the left portal vein and right portal vein; umbilical cord contains four vessels	Better prognosis for isolated cases; severity of additional anomalies impacts outcome	Extended fetal anatomical evaluation recommended. No follow-up needed for isolated cases. For additional findings, genetic and invasive testing should be considered
Umbilical vein varix [32,33]	The umbilical vein appears as a dilated segment with anechoic structure; bidirectional, turbulent flow detected on Doppler	Low risk of complications in isolated cases	No follow-up required for isolated cases. Genetic testing advised for non-isolated umbilical vein varix cases
Umbilical vein constriction [34-36]	Narrower umbilical vein diameter; high-speed blood flow detected (150-200 cm/s)	Associated with intrauterine fetal demise, intrauterine growth restriction, and oligohydramnios	Requires close fetal ultrasound monitoring

The Relationship With Hepatic Surgery and Umbilical Vein Interventions

Prior to extended liver surgery, selective portal vein embolization is a well-accepted method for stimulating future liver remnant growth. An uncommon but serious complication of portal vein embolization is portal vein thrombosis involving the main stem and non-targeted branches supplying the future liver remnant. This condition necessitates urgent revascularization to restore blood flow. Without timely intervention, curative liver surgery becomes unfeasible, posing a potentially life-threatening situation for the patient. The study by Derksen et al. [37] introduces a novel surgical technique for revascularizing the portal vein following portal vein thrombosis. This approach combines surgical thrombectomy with catheter-based thrombolysis through the surgically reopened umbilical vein. The technique was successfully applied in a patient who developed thrombosis in the portal vein main stem, the left portal vein, and its branches to the left lateral segments after selective right-sided portal vein embolization in preparation for an extended right hepatectomy. A key advantage of this method is that it avoids exploration of the hepatoduodenal ligament and venotomy of the portal vein, thus minimizing surgical trauma. This facilitates the use of additional intravascular thrombolytic therapy and supports the planned extended right hepatectomy. The technique is particularly recommended for patients with extensive portal vein thrombosis where less invasive percutaneous therapies have been ineffective [37].

Recent advancements in understanding the anatomical characteristics of biliary structures have facilitated the increased use of pedicled umbilical vein patch (PUVP) repair, typically performed under laparotomy [38]. However, when compared to the open approach, laparoscopic suturing, especially for end-to-end anastomosis, can be challenging due to limited visualization and movement. Regardless, this technique is feasible and beneficial for the patient. More than 4000 laparoscopic operations have been performed since its establishment. Liao et al. [39] highlighted the laparoscopic repair of left hepatic duct stenosis using PUVP in patients with hepatolithiasis. Their findings suggest that this laparoscopic technique is both safe and feasible, offering advantages such as reduced trauma, faster recovery, and less postoperative discomfort compared to traditional laparotomy. This approach represents a promising alternative for managing biliary duct stenosis with minimized invasiveness in comparison to laparotomy.

Another common practice in newborn medicine is the insertion of umbilical cord catheters (UCCs). They play a crucial role in the primary care of critically ill preterm and term infants. Their indications are well established, including resuscitation, drug administration, and secure vascular access. UCCs can be inserted through either the umbilical artery or vein, depending on specific contraindications. The umbilical vein follows a short subcutaneous course before reaching the anterior inferior margin of the liver. After making a posterior turn, it terminates in the left branch of the portal vein, forming a slight expansion known as the umbilical recess. From this recess, segmental portal veins (SPV) branch out to supply other liver lobes [40]. When placing an umbilical vein catheter (UVC), it is inserted into the single umbilical vein until it reaches the umbilical recess and, if correctly positioned, should extend just into the inferior vena cava via the ductus venosus. The ductus venosus originates directly from the umbilical recess, opposite the umbilical vein, and courses dorsally through the liver before draining into the inferior vena cava at the confluence of the hepatic veins. This direct anatomical connection between the umbilical vein and inferior vena cava facilitates the passage of oxygenated blood into the systemic circulation. There is significant variability in reported rates of correct UCC positioning, ranging from 28% to 75% [41]. Malpositioning of the UVC into the hepatic veins is particularly common, often resulting in catheter dysfunction. Furthermore, anatomical variations can make the placement of UCC particularly challenging. Thus, being accustomed to such altered structures is key to achieving a proper intervention for the patient.

Discussion

The formation of the rudimentary umbilical cord begins between the fourth and eighth weeks of gestation. This process involves the envelopment of tissue from the body stalk, the omphalomesenteric duct, and the umbilical coelom by the expanding amnion. Notably, blood flow within the umbilical cord is established by the end of the fifth week of gestation, marking a pivotal moment in fetal development [1]. The umbilical cord eventually contains one umbilical vein and two umbilical arteries, embedded in Wharton's jelly and encased by a layer of amnion. The umbilical cord serves as the lifeline between the fetus and the placenta, carrying oxygenated blood through the umbilical vein and returning deoxygenated blood via the umbilical arteries. The length and diameter of the umbilical cord can have significant clinical implications. Abnormal lengths can lead to complications such as prolapse, entanglement, and fetal distress [8]. The development of the umbilical cord's vessels also has postnatal significance, with remnants of the umbilical arteries and vein contributing to structures such as the medial umbilical ligaments and the ligamentum teres [3].

Early in embryonic development, both umbilical veins are connected to the sinus venosus, but by the seventh week of gestation, the right umbilical vein is typically obliterated, leaving the left umbilical vein as the primary vessel for carrying blood from the placenta to the fetus. However, anomalies, such as the persistence of the right umbilical vein and duplication of the umbilical vein, can occur, affecting the normal flow of oxygenated blood. These anomalies can range from being asymptomatic to being associated with severe fetal malformations, particularly when linked with abnormalities in the ductus venosus [20]. The incidence of a persistent right umbilical vein, while uncertain, is thought to be more common than previously believed, with diagnoses occurring in approximately 1/250 to 1/1250 pregnancies [28]. This anomaly underscores the variability of umbilical vein development and the potential for overlooking abnormalities during standard prenatal ultrasounds. Advanced imaging techniques, such as color Doppler and three-dimensional ultrasounds, improve the detection and understanding of such conditions [29]. The presence of the persistence of the right umbilical vein or duplication of the umbilical vein does not necessarily entail adverse outcomes, especially when these anomalies exist in isolation, without other malformations. However, their association with congenital malformations, particularly when there is a congenital absence of the ductus venosus, necessitates careful prenatal monitoring to detect any signs of hemodynamic compromise. Umbilical vein varix and umbilical vein constriction are additional umbilical vein abnormalities with significant clinical implications. Umbilical vein varix, characterized by the significant dilation of the umbilical vein, and umbilical vein constriction, marked by a narrowed diameter and potentially reduced blood flow to the fetus, can lead to critical outcomes, including IUGR, fetal hypoxia, acidosis, and IUFD [32]. The early identification of these conditions through fetal surveillance is vital to prevent adverse perinatal outcomes. Additionally, studies show that the abnormal variations of the course of the umbilical vein and its ligamentous sheath can lead to anomalies in the infant as well as in the adolescent [24]. Few cases have reported obstructive ileus due to internal herniation or compression by the abnormally positioned umbilical vein remnant within the peritoneal cavity. It is important for medical doctors and specifically surgeons to be accustomed to such variations and their severe outcomes in order to plan their treatment approach accordingly.

The primary diagnosis of umbilical vein anomalies in fetuses involves specific sonographic criteria, including the course of the portal vein, the position of the fetal gallbladder relative to the umbilical vein, and the vein's connection to portal vessels. It is of vast importance that early detection and accurate diagnosis are crucial for managing potential complications associated with these anomalies [35].

A variety of novel interventions have arisen by studying the embryological remnants of the umbilical vein. Selective portal vein embolization is a well-recognized preoperative procedure designed to stimulate growth in the future liver remnant. However, one of the severe complications of portal vein embolization is portal vein thrombosis, which can compromise the viability of the future liver remnant and render curative liver surgery unfeasible. Derksen et al. presented a new technique combining surgical thrombectomy by means of catheter-based thrombolysis through the surgically recanalized umbilical vein [37]. This approach offers a minimally invasive alternative to traditional methods, avoiding the exploration of vital structures of the hepatoduodenal ligament. The lesser tissue trauma associated with this technique not only allows for the effective management of portal vein thrombosis but also facilitates the administration of additional intravascular thrombolytic therapy, thereby preserving the feasibility of subsequent liver resection. Another novel technique that has been used recently is the application of PUVP repair in cases of biliary strictures. This procedure is highly promising for faster recovery and reduced trauma for patients. Both the revascularization technique for portal vein thrombosis and the laparoscopic PUVP repair for hepatic duct stenosis exemplify the dynamic nature of surgical innovation in liver surgery [38].

For hepatobiliary surgeons, comprehensive anatomical knowledge of these rare anomalies is of paramount significance. Surgeons must be well versed in both normal and variant embryological development to accurately diagnose and manage these anomalies, especially during exploratory laparotomy or minimally invasive procedures. Failure to recognize such variations may lead to misdiagnosis, prolonged operative time, or inadvertent damage to critical structures. For that reason, preoperative imaging studies, including high-resolution ultrasound, contrast-enhanced CT scans, MR angiography, and MR venography, are invaluable in identifying anomalies before surgery. In particular, three-dimensional reconstructions from CT or MRI data can provide detailed vascular details, allowing surgeons to visualize atypical vascular courses, whether the presence of an aberrant ligamentum teres or a persistent right umbilical vein, enhancing surgical precision and reducing intraoperative surprises. Additionally, advancements in minimally invasive techniques, such as laparoscopic and robotic-assisted surgery, allow for enhanced visualization and precise dissection of aberrant structures with minimal tissue trauma. Robotic platforms, in particular, offer the advantage of magnified three-dimensional visualization and articulate instruments that mimic the dexterity of open surgery, thus facilitating safer navigation around anomalous vessels. As these techniques become increasingly refined and more widely adopted, operative times may be reduced, postoperative pain minimized, and hospital stays shortened. Emerging technologies, such as intraoperative fluorescence angiography or hybrid operating room setups with real-time imaging, can further refine the intraoperative identification and protection of vascular structures.

## Conclusions

Studying and understanding the different variations of the umbilical vein during development and throughout an adult's life is important. Some of these abnormalities may be potentially fatal or pose a severe threat to the patient. These variations can appear as silent or in some cases symptomatic. Therefore, in clinical terms, early and accurate detection of these malformations is highly valued for the sake of a proper diagnosis and management of care. The combination of certified surgeons and novel and promising techniques that are being established are paramount in order to achieve optimal care for such patients.
